# Pathophysiology of Coronary Thrombus Formation and Adverse Consequences of Thrombus During PCI

**DOI:** 10.2174/157340312803217247

**Published:** 2012-08

**Authors:** Sundararajan Srikanth, John A Ambrose

**Affiliations:** 1 Interventional Cardiology Fellow, UCSF Fresno, University of California San Francisco Chief of Cardiology, UCSF Fresno; 2 Professor of Medicine, University of California San Francisco Chief of Cardiology, UCSF Fresno

**Keywords:** Coronary thrombus, percutaneous intervention, endothelial dysfunction, atherosclerosis.

## Abstract

Atherosclerosis is a systemic vascular pathology that is preceded by endothelial dysfunction. Vascular inflammation “fuels” atherosclerosis and creates the milieu for episodes of intravascular thromboses. Thrombotic events in the coronary vasculature may lead to asymptomatic progression of atherosclerosis or could manifest as acute coronary syndromes or even sudden cardiac death. Thrombus encountered in the setting of acute coronary syndromes has been correlated with acute complications during percutaneous coronary interventions such as no-reflow, acute coronary occlusion and long term complications such as stent thrombus. This article reviews the pathophysiology of coronary thrombogenesis and explores the complications associated with thrombus during coronary interventions.

## INTRODUCTION 

Extensive research in vascular biology over the past few decades has substantially advanced our knowledge of the pathophysiology of atherosclerosis and athero-thrombosis. The role of the endothelium in maintaining vascular health and the link between endothelial dysfunction and atherosclerosis has been explored. The systemic nature of endothelial dysfunction leading to localized manifestations of atherosclerosis, exacerbated by inflammation is now appreciated. The clinical manifestations of acute coronary syndromes and chronic ischemic coronary artery disease are ultimately a consequence of these processes. The evolution of the acute coronary syndromes is particularly related to the development of intravascular athero-thrombotic disease involving the coronary vasculature. The recognition of these thrombogenic processes and their sequelae is essential to the management of these syndromes.

## ENDOTHELIUM, ENDOTHELIAL DYSFUNCTION AND ATHEROSCLEROSIS 

The mono-cellular layer of endothelial cells which lines the vascular lumen is critical in maintaining normal vascular flow. The endothelium modulates vascular flow by controlling vasodilator tone and it inhibits platelet aggregation and clotting factor activation by providing a barrier to the procoagulant sub-endothelial tissue. A healthy endothelium also acts as a barrier to inflammation and is able to adequately repair itself after injury. Endothelial dysfunction is manifested by vasoconstriction, thrombosis, inflammation and smooth muscle proliferation. Atherosclerotic lesions seem to develop under an intact but leaky and dysfunctional endothelium. Many traditional coronary risk factors that predispose to atherosclerosis such as hypercholesterolemia, hypertension and a positive family history are associated with endothelial dysfunction [1-3]. Prospective cohort studies have shown endothelial dysfunction to independently predict progression of atherosclerosis and acute cardiovascular events in patients with and without known coronary artery disease [4,5].

**Fig. (1):**
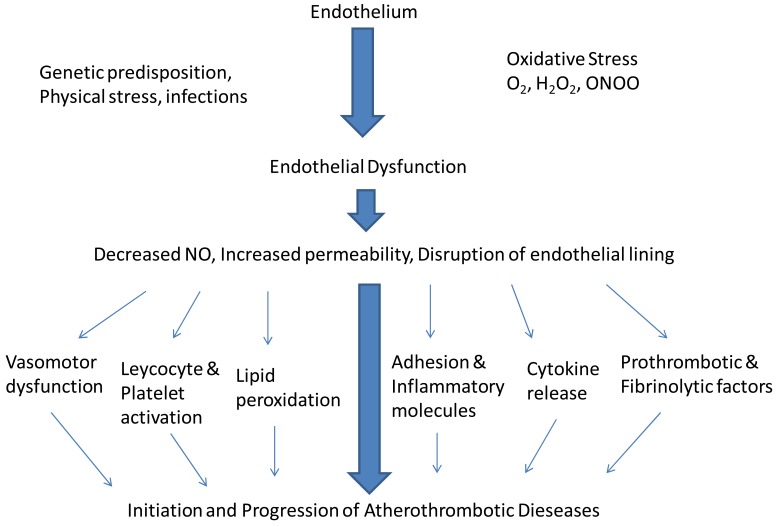
Endothelial dysfunction and atherosclerosis.

Ludmer *et al*. provided the first evidence of endothelial dysfunction in humans [6]. They showed paradoxical constriction of the coronary arteries to acetylcholine in individuals with mild as well as severe coronary artery disease, suggesting that endothelial dysfunction is present early in the development of atherosclerosis. Endothelial dysfunction is thought to result from damaging environmental exposure possibly exacerbated by genetic predisposition. Oxidative injury from various metabolic derangements in the local environment, physical forces (shear stress) and even infectious processes may all contribute to endothelial injury. Endothelial dysfunction results in a leaky endothelial lining which allows for passage of pro-atherogenic stimuli into the sub-endothelial space. Later, endothelial cells may vanish and denuded areas appear with exposure of blood to sub-endothelial tissue [7]. Loss of endothelial function leads to reduced local nitric oxide availability along with increased expression of prothrombotic factors, chemokines and proinflammatory mediators and reduced number and function of endothelial progenitor cells [8]. With lower nitric oxide availability there is increased expression of intercellular adhesion molecules by the endothelial cells which leads to the binding of monocytes and lymphocytes, with subsequent invasion of the vascular wall [9]. The complex local interactions surrounding the damaged endothelium facilitates further passage of lipids and leucocytes into the sub-endothelium. Monocytes transmigrate into the subendothelial space, ingest the oxidized lipoproteins and then are transformed into macrophages. Macrophages accumulate oxidized LDL transforming into foam cells. These foam cells form the initial lesions leading to advanced atherosclerosis [10,11] (see Fig. **1**).

## STABLE AND “VULNERABLE” PLAQUES 

**Fig. (2):**
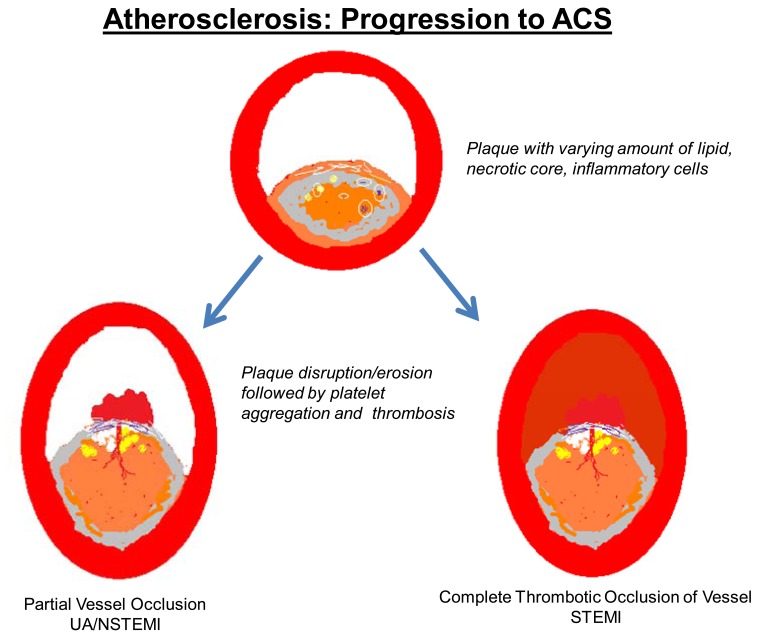
Atherosclerosis: progression to ACS.

Foam cells secrete pro-inflammatory cytokines including growth factors, matrix metalloproteinases and tissue factor leading to migration and proliferation of smooth muscle cells in the lesions [10]. Recurrent bouts of inflammation induce further smooth muscle cell proliferation and migration into the intima. This can lead to the transformation into a complex lesion consisting of inflammatory cells, smooth muscle cells, and intracellular and extracellular lipid [11, 12]. Neovascularization of these lesions with immature vasculature may lead to hemorrhage within the lesions further increasing plaque size and lipid content [13-16]. Generally, a thick layer of smooth muscle cells, collagen and elastin forms a cap that covers the lesion and keeps its contents sequestered from the blood stream. These voluminous lesions cause varying degrees of remodeling of the vessel wall along with varying degrees of stenosis of the intraluminal area. Inflammation leads to worsening atherosclerosis and thinning of the fibrous cap with the potential for subsequent plaque destabilization (Fig. **2**).

The balance between cell migration, cell proliferation, extracellular matrix formation, inflammatory leucocyte interaction, and cell apoptosis plays a role in the transition from a stable plaque to a potentially high risk or so called “vulnerable” plaque. As to which plaque is likely to become the site of an intraluminal coronary thrombus has been intensively studied and debated [17]. Digestion of the fibrous matrix by matrix metallo-proteinases and apoptosis of the smooth muscle cells forming the caps may lead to weakening and thinning of the fibrous cap. The death of macrophages by apoptosis and necrosis along with plaque hemorrhage from leaky vasa-vasorum contributes to the formation of a soft and destabilizing lipid-rich core within the plaque. Macrophages and T-cells congregating at sites of fibrous cap disruption indicate their key role in plaque destabilization releasing necrotic material into the vessel lumen along with cholesterol crystals. The necrotic sub-endothelial material along with lipid crystals interacts with platelets in the blood leading to coronary thrombus formation. In addition, tissue factor that is exposed with plaque disruption results in activation of the extrinsic coagulation pathway.

Following plaque disruption, the plaque contents come in contact with blood. Freely circulating platelets in circulation promptly adhere to the sub-endothelial matrix and damaged endothelial cells. Adhesion of platelets is mediated by the binding of surface glycoproteins to endothelial ligands. Platelet surface glycoprotein GP Ib/IX recognizes von-Willebrand factor synthesized and stored in endothelial cells. Platelet glycoprotein GPIa/IIa binds collagen present in the deeper vessel wall. Following adhesion, activated platelets release multiple intermediaries including serotonin, ADP, thromboxane A2, endothelin, free radicals and platelet activating factor which promote further platelet aggregation and vasoconstriction. These platelet aggregates forming a so called white thrombus tend to be unstable and may cause intermittent reduction in blood flow and distal embolization [18]. The coagulation status of blood is a significant determinant of the end result of plaque disruption. Rapid activation of the coagulation system with fibrin deposition strengthens the platelet aggregates causing persistent obstruction to flow. This along with impaired flow dynamics from vasoconstriction leads to pooling of blood and formation of fibrin rich red thrombi [19].

## CLINICAL MANIFESTATIONS OF ATHERO-THROMBOTIC PROCESSES IN CORONARY ARTERIES 

**Fig. (3):**
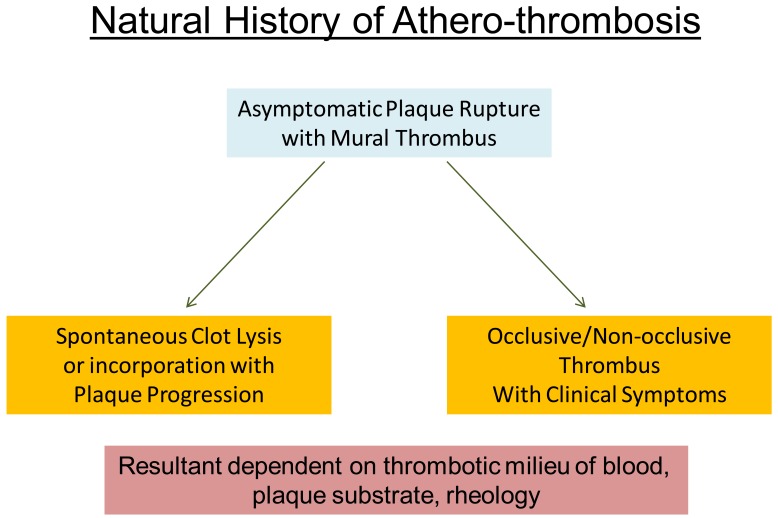
Natural history of athero-thrombosis.

The atherosclerotic processes involving the vasculature evolve over many years (Fig. **3**). Early plaque formation is often associated with outward remodeling of the vessel. When this adaptive process is exceeded, the plaque starts to encroach into the lumen of the vasculature. It is now recognized that thrombosis from plaque disruption or erosion of a large plaque that is not severely occlusive prior to the event is most often the immediate cause of acute coronary syndromes, particularly ST elevation myocardial infarction [20]. Plaque disruption is found at pathology in 10% of individuals with atherosclerosis dying of non-cardiac disease. Conversely, thrombi are frequently observed at sites other than those of the major culprit lesion in patients dying of acute coronary syndromes [21]. Thus, thrombus formation on a plaque may or may not lead to a clinical syndrome. While plaque disruption with thrombus formation is thought to be the major pathogenetic mechanism for acute coronary syndromes, the vast majority of plaque fissures are asymptomatic and may only contribute to the slow progression of atherosclerotic lesions [22]. While plaques associated with acute myocardial infarction are usually large, expansively remodeled and not severely narrowed prior to the clinical event, plaques responsible for stable angina usually are smaller but, often are associated with more severe luminal narrowing because of concomitant constrictive remodeling [23-25].

About 70% of cases of acute coronary thrombosis involve a disrupted atherosclerotic plaque and in the remaining 30%, there is only superficial intimal injury at the site of thrombus formation [26]. Superficial endothelial erosion leading to a clinically evident coronary thrombosis is most commonly seen in women and in diabetics with hypertriglyceridemia. While the exact mechanism of superficial erosion is not clear, it is likely that matrix metallo-proteinases in the subendothelium may disrupt the tethering of the endothelial cell to the basal lamina leading to desquamation [27]. Many cases are likely associated with a prothrombotic milieu. Most episodes of endothelial erosion are also likely asymptomatic. However erosion may lead to non-occlusive thrombus formation followed by healing. Such repetitive cycles could contribute to slow atherosclerotic progression.

## DETERMINANTS OF THE ACUTE CLINICAL SYNDROME 

**Fig. (4):**
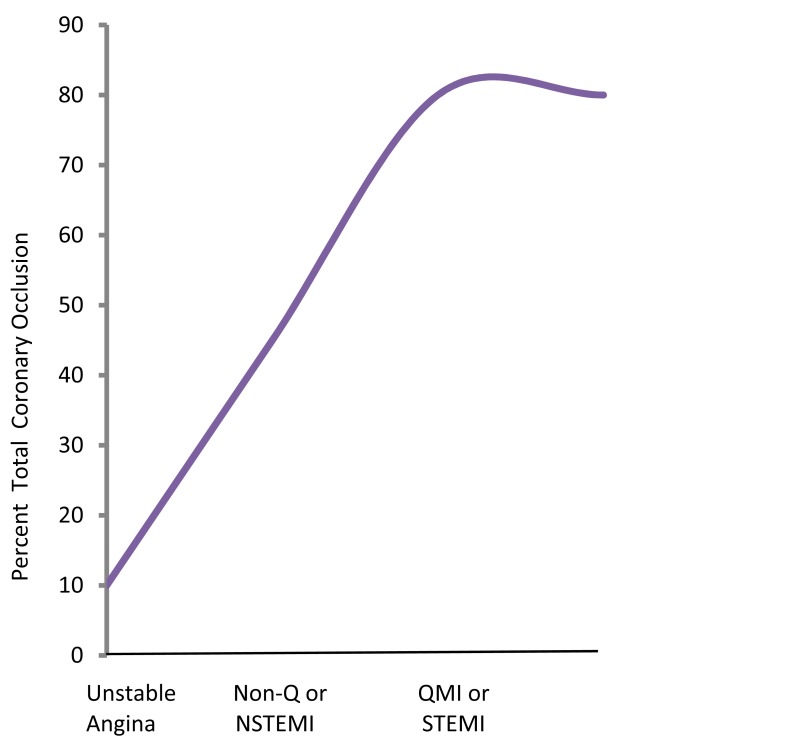
Correlation of clinical symptomatology with extent of coronary occlusion. There is a range of complete or total coronoary occlusion dependent on the acute syndrome. In unstable angina it ranges upto 20%, in NSTEMI from 20-40% and in STEMI upto 85-90%. – *after De-Wood 1980 & Ambrose 1985, 1986*

Apart from the characteristics and volume of the plaque content and its proximal location in the coronary tree, the cellular and humoral components of blood, the extent and type of thrombus formation at the site of plaque disruption/erosion and myocardial vulnerability in the individual contribute to the extent and type of clinical manifestation if any. The syndrome ultimately developed also depends on factors such as the degree and acuteness of obstruction, the duration of decreased perfusion and the relative myocardial oxygen demand as well as the collateral circulation [28]. Angiographic, biochemical, pharmacologic and surgical data support the role of thrombus formation in patients presenting with STEMI, NSTEMI and unstable angina [20, 29]. When unstable angina is defined as the new onset of low work load or rest angina or an abrupt change in angina that had previously been stable, the incidence of finding a complex plaque (i.e. an eccentric stenosis with overlapping edges, irregular borders, ulcerations and/or filling defects in the culprit vessel) is approximately 70% on angiographic analysis [30]. In patients with a short duration of unstable angina or with very recent onset of rest pain, the incidence of thrombus has been reported to be even higher [31]. Coronary angioscopy has demonstrated an even higher incidence of mural thrombus in patients presenting with unstable angina [32, 33]. In unstable angina and non-Q wave myocardial infarction (the forerunner of NSTEMI), the thrombus is more likely to be non-occlusive than in an evolving Q-wave myocardial infarction or STEMI, where the thrombus in the first few hours after infarction is occlusive in greater than 80% [29] (Fig. **4**). Not all patients with unstable angina/NSTEMI necessarily have plaque disruption/erosion. Type II myocardial infarction related to a supply/demand mismatch [34] accounted for about 30% of all myocardial infarction in a prospective study by Javed *et al* [35].

## CORONARY THROMBUS AND PCI 

**Fig. (5):**
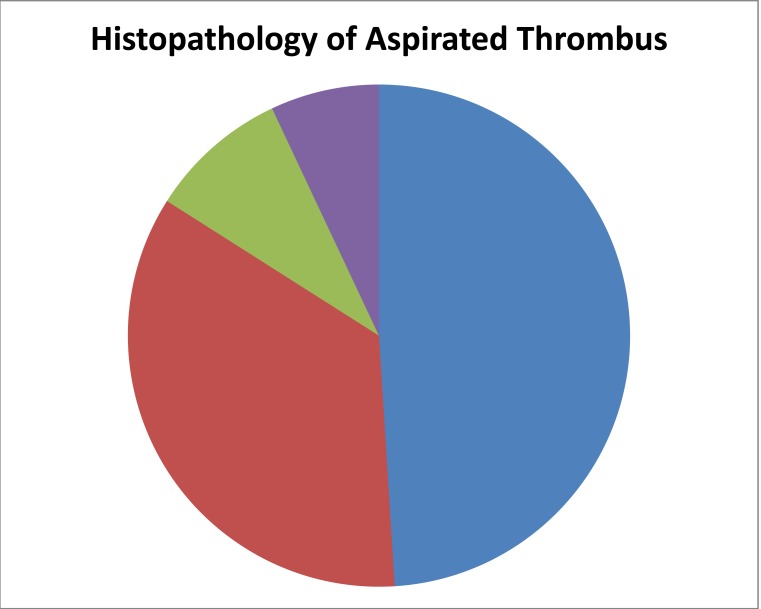
Histopathology of aspirated thrombus. Adapted from Rittersma et al. Circulation 2005; 111: 1160-1165.

Pathological findings of thrombus collected by thrombectomy procedures during PCI in the last few years corroborate the role of in situ thrombus in acute coronary syndromes. Histopathological analysis of aspirated thrombotic content show erythrocyte-rich (red) thrombus in about 35% of patients, predominantly in those presenting with low TIMI flow. A platelet-rich thrombus is identified in 65% of cases, particularly in the early hours of acute myocardial infarction [36]. It has also come to light that the composition of thrombus is often heterogenous. The composition of the isolated thrombus often shows fresh thrombus along with features of organization, and lytic changes in the same tissue fragment [37]. The layered composition of clots suggests episodic growth of thrombus for a finite interval before the onset of occlusive thrombus and clinical symptomatology. Analysis of electron microscopic images of thrombus obtained from thrombectomy procedures shows that formation of the thrombus is dynamic and that the composition of the thrombus varies with the ischemia time. Fresh thrombi have a highest proportion of platelets, whereas the proportion of fibrin fibers increases over time leading to older more fibrin rich thrombi [38] (Fig. **5**).

The thrombus burden associated with an acute coronary syndrome may vary depending on various factors, including size of the vessels, duration of occlusion, prothrombotic state etc. Some of the clinical risk factors associated with higher thrombus burden include hypercholesterolemia, smoking and male gender [39]. In addition, a variety of local factors affect the genesis and behavior of thrombus generated in situ including interaction between platelets, vessel wall, red blood cells, plaque gruel and coagulation proteins. The behavior of thrombus during percutaneous intervention may be quite variable and may influence the outcome of the intervention. The extent and duration of fibrin polymerization and stabilization in an evolving thrombus may contribute to the differing behavior of thrombus including the tendency for friability during catheter or wire maneuvering despite rigid adherence to the underlying plaque.

The fibrin network in thrombi, when examined by electron microscopy shows two distinct types of patterns [40]. One pattern consists of dense scaffolding of thin fibers that is resistant to mechanical force and thrombolysis as compared to the second pattern consisting of thick loosely packed fibers that are more susceptible to thrombolysis. Interactions between platelets, red blood cells, vessel wall, fibrinogen and other local chemicals in the local environment in addition to the age of the thrombus may all have an impact on the fibrin network and thus on the strength and behavior of a thrombus. The characteristics of the clots as seen on angiography correlate with the histology of extracted thrombus [41, 42]. The slightly altered behavior of coronary thrombus in the setting of tobacco use might be explained by differences in the fibrin architecture as seen by electron microscopy [43].

Other factors that can influence clot burden and behavior include characteristics of the underlying vessel; notably coronary arteries with ectasia, vasculitis and aneurysms are more likely to have large thrombi due to stasis as do saphenous vein grafts. Late presentation with an established myocardial infarction and cardiogenic shock, inadequate anti-coagulant or anti-platelet therapy and complications related to therapy such as HIT may also increase thrombus burden. The right coronary artery tends to have a larger burden of thrombus probably because of proximal propagation of thrombus related to fewer branch points. Hyperglycemia and leukocytosis may also accentuate thrombosis. During PCI, thrombus growth may be triggered by guidewires, stasis of blood, inadequate antithrombotic/anticoagulant therapy, balloons or stent; the “angry clot” phenomenon [44, 45].

## COMPLICATIONS OF PCI RELATED TO INTRACORONARY THROMBUS 

Prior to the introduction of stents and methods to extract or dissolve thrombus, balloon angioplasty of thrombotic lesions posed many procedural difficulties for the interventionalist including major dissection, vasoconstriction, abrupt closure and total occlusion with the need for emergent bypass and associated with an increased mortality [46,47]. Pre-existing thrombus was also shown to increase angiographic restenosis, mainly through early vessel occlusion [48]. Pre-existing thrombus continued to be an independent predictor of angioplasty failure until the introduction of stents. While many of these procedural complications have been significantly reduced in the stent era, there continue to be adverse complications of intracoronary thrombus during PCI and stenting related to embolization, the “no reflow” phenomenon and stent thrombus. The limited success of standard PCI even with stents, in lesions with a large thrombus burden despite the illusion of adequate reperfusion is clinically relevant and contributes to the no-reflow phenomenon [49]. Of note, in some studies the desired grade 3 myocardial blush score was gained only in a minority of patients in whom TIMI 3 flow was established with standard PCI at the time of primary PCI [50].

The first large scale study of the effect of thrombus burden during PCI was reported by Harjai *et al* [51]. About 6% of patients in the study that included 2,148 subjects from PAMI-2, Stent PAMI and PAMI No-Surgery-On-Site trials had angiographically visible thrombus after PCI. The presence of thrombus before PCI was independently associated with a higher incidence of post-PCI thrombus likely reflecting a larger thrombus burden prior to intervention. Both the thrombus burden and thrombus characteristics play a role in contributing to the no-reflow phenomenon. The higher thrombus burden can lead to more distal embolization obstructing flow within distal arterial segments and microvessels leading to no-reflow [52]. Yip *et al*. proposed a score to assess thrombus burden on the basis of angiographic features (Table **3**) [53].

All of these features were independent predictors of no-reflow in 800 patients undergoing primary PCI. Using Yip’s score, Limbruno *et al* were able to predict total debris volume captured by distal filter wire in patients undergoing PCI for STEMI [54]. Patients with no-reflow following PCI for STEMI have also been shown to have decreased clot permeability with increased resistance to lysis of thrombus when compared to patients with more normal flow [55]. Of note, distal embolization of thrombotic debris often occurs after stent deployment in large coronary vessels, whereas in small vessels it is possible that the stent itself might fix the thrombus to the vessel wall.

Patients who develop no-reflow related to distal embolization sustain larger infarcts leading to poorer outcomes [56]. Distal embolization occurs in upto 15% of patients undergoing PCI and is associated with as much as a seven fold increase in the rate of periprocedural MI. In more recent trials with concurrent use of anticoagulants and dual antiplatelet therapy the incidence of distal embolization is approximately 6% [57]. These complications can be seen more often in patient undergoing PCI for acute myocardial infarction [58]. Individuals with angiographically evident embolization have significantly worse outcomes than patients without, as expressed by lower myocardial blush grade (MBG), impaired ST-segment resolution, and higher level of myocardial enzyme leakage [59-64]. Thrombus embolization also significantly increases the need for emergency bypass surgery as well as the procedure-related death rate [65].

No-reflow in the setting of a large thrombus can be assessed with TIMI flow grade and MBG during PCI. Clinically it can be suspected by lack of ST segment resolution. Noninvasive imaging techniques such as myocardial contrast echocardiography and cardiac magnetic resonance imaging provide a more accurate assessment of myocardial perfusion and hence a better assessment of no-reflow following PCI [66,67]. Direct stenting has been suggested as a technique to reduce distal embolization, by avoiding balloon-induced thrombus fragmentation and entrapment of thrombus under the stent struts [68]. However, direct stenting is feasible only in patients with good distal visualization of infarct related artery after passage of a guidewire.

## STENT THROMBOSIS 

The etiology of stent thrombosis is multifactorial, and includes stent thrombogenicity, in addition to procedure related, lesion related, and patient-related factors [69]. Randomized trials of BMS implantation during elective PCI have reported stent thrombosis rate of 0.4 to 1.3% [70,71]. Results from a pooled analysis of 10 randomized controlled trials of elective PCI reported similar rates of stent thrombosis between BMS and DES of around 0.6%; but most studies indicate a higher rate of very late stent thrombosis with DES compared to BMS, particularly for the first generation DES [72-75]. Stent underexpansion, malapposition, residual dissections, and inflow/outflow disease have been well established by intravascular ultrasound as mechanical causes related to early stent thrombosis for both BMS and DES [76-79]. However, by and far premature discontinuation of dual anti-platelet therapy remains the most frequent but not the only cause of stent thrombosis [80].

Apart from mechanical factors related to the stents, there are other variables including alterations in the coagulation cascade and response to pharmacotherapy that may influence the behavior of thrombus during PCI, particularly in the setting of STEMI. Acute coronary syndromes by their very evolution have intracoronary thrombus and have been associated with higher rates of stent thrombosis following PCI. In the TYPHOON (Trial to Assess the Use of the Cypher Stent in Acute Myocardial Infarction Treated With Angioplasty) trial, which randomized STEMI patients to SES or BMS, the angiographic stent thrombosis rate was 2.0% and 3.4%, respectively, at 1 year [81]. Clearly inadequate anticoagulation and antiplatelet therapy during PCI is associated with increased thrombus burden during PCI and consequent stent thrombosis. An impaired response to antiplatelet therapy may also predispose to a large thrombus burden and consequent stent thrombus, particularly in the setting of ACS [82,83]. The impaired response may be in the form of resistance to either aspirin, clopidogrel or side effects of medications such as in the case of heparin- induced thrombocytopenia.

Along with other causes, the presence of thrombus has been identified as a factor predisposing to stent thrombosis [69]. Thrombus that is compressed against the vessel wall and or displaced by stent struts during primary PCI may cause problems in the long term due to resolution of thrombus leaving behind a malapposed stent. Thus a larger thrombotic burden during PCI may increase the risk of sent thrombosis due to late malapposition. This was shown by Sianos *et al* in a retrospective cohort of 812 consecutive patients presenting with STEMI who had primary PCI with use of a drug eluting stent [84]. A large thrombus burden was a fundamental factor for adverse clinical outcomes including increased 30 day mortality, with high rates of infarct-related stent thrombosis accounting for majority of the post-30-days MACE rate. Large thrombus burden in that study was defined as a filling defect whose length was greater than or equal to twice the vessel diameter. In patients with total occlusion of the infarct artery, reperfusion was established with a wire or small balloon before thrombus burden was assessed; this was an important aspect of the methodology since a simple classification based on TIMI flow would have excluded a majority of the subjects who would have presented with TIMI flow of zero. The authors found that the risk of subsequent stent thrombosis after primary PCI with stenting can be dramatically reduced with rheolytic thrombectomy.

In summary intra-coronary thrombogenesis is a dynamic process and the thrombus burden in the coronary vasculature during an acute coronary event is variable. The factors that influence the burden and behavior of the thrombus are complex. Elaboration of these processes has led to the recognition and improved anticipation of their consequences.

## CONCLUSION 

Endothelial dysfunction and atherosclerosis set the stage for coronary artery disease. Acute clinical manifestations occur from destabilization of atherosclerotic plaques within the coronary vessels, most often secondary to intraluminal thrombus formation. Appreciating the role of thrombus formation in ACS has lead to a significant evolution in the management of these challenging patients. Thrombus during percutaneous intervention poses a formidable challenge for the interventionalist both in terms of dealing with the possibility of embolization and no-reflow in the acute situation and stent thrombus acutely and in the long term. Though thrombus may be detected by various imaging modalities including angiography, ultrasound, and OCT, angiographic techniques have been standardized and still remain the initial modality routinely used for decision making during PCI especially in the setting of STEMI and other NSTEACS. Several techniques both pharmacologic and mechanical have been developed to deal with intra-coronary thrombus. A general understanding of the processes involved in atherothrombosis and its treatment is essential in maximizing patient outcomes.

## Figures and Tables

**Table 1.** Factors Associated with Thrombus Behavior

Anticoagulant and antiplatelet therapies	Inflammation/Leukocytosis
Duration of occlusion	Plaque contents
Fibrin architecture	Platelet activation
Gender	Tobacco use
Hypercholesterolemia	Vessel size
Hypercoagulable states	

**Table 2.** Sequelae of Intracoronary Thrombus During PCI

Recurrent Myocardial Infarction	Stent Thrombosis
Due to embolization	Early
From acute closure	Late
No-reflow	

**Table 3.** Assessment of Thrombus Burden by Angiography

Thrombus with greatest linear dimension more than three times the reference lumen diameter
Cutoff pattern
Presence of accumulated thrombus (>5mm of linear dimension) proximal to the occlusion
Presence of floating thrombus proximal to the occlusion
Persistent contrast medium distal to the obstruction
Reference lumen diameter of the infarct related artery greater than 4 mm

- Adapted from Yip et al. Chest 2002; 122: 1322-32
